# Positive modulation of a new reconstructed human gut microbiota by Maitake extract helpfully boosts the intestinal environment *in vitro*

**DOI:** 10.1371/journal.pone.0301822

**Published:** 2024-04-11

**Authors:** Alessandra De Giani, Federica Perillo, Alberto Baeri, Margherita Finazzi, Federica Facciotti, Patrizia Di Gennaro

**Affiliations:** 1 Department of Biotechnology and Biosciences, University of Milano-Bicocca, Milano, Italy; 2 Department of Experimental Oncology, European Institute of Oncology, Milan, Italy; University of Illinois Urbana-Champaign, UNITED STATES

## Abstract

The human gut is a complex environment where the microbiota and its metabolites play a crucial role in the maintenance of a healthy state. The aim of the present work is the reconstruction of a new *in vitro* minimal human gut microbiota resembling the microbe-microbe networking comprising the principal phyla (Bacillota, Bacteroidota, Pseudomonadota, and Actinomycetota), to comprehend the intestinal ecosystem complexity. In the reductionist model, we mimicked the administration of Maitake extract as prebiotic and a probiotic formulation (three strains belonging to *Lactobacillus* and *Bifidobacterium* genera), evaluating the modulation of strain levels, the release of beneficial metabolites, and their health-promoting effects on human cell lines of the intestinal environment. The administration of Maitake and the selected probiotic strains generated a positive modulation of the *in vitro* bacterial community by qPCR analyses, evidencing the prominence of beneficial strains (*Lactiplantibacillus plantarum* and *Bifidobacterium animalis* subsp. *lactis*) after 48 hours. The bacterial community growths were associated with the production of metabolites over time through GC-MSD analyses such as lactate, butyrate, and propionate. Their effects on the host were evaluated on cell lines of the intestinal epithelium and the immune system, evidencing positive antioxidant (upregulation of SOD1 and NQO1 genes in HT-29 cell line) and anti-inflammatory effects (production of IL-10 from all the PBMCs). Therefore, the results highlighted a positive modulation induced by the synergic activities of probiotics and Maitake, inducing a tolerogenic microenvironment.

## 1. Introduction

Nowadays, at least 2,786 mushroom species are consumed in 99 countries [1] because of their high nutritional value due to the presence of essential nutrients [2]. Among them an interesting fungus is *Grifola frondosa*, known also as Maitake [3]. It is characterized by flavonoids, organic acids, alkaloids, and several polysaccharides, such as long-chain β-glucans with β(1–3), β(1–4), and β(1–6) glycosidic linkages [4, 5]. These edible mushroom polysaccharides (EMPs) have outstanding physiological effects, comprising immunomodulatory, antioxidant, and anti-inflammatory activities on the gastrointestinal tract [4, 6]. EMPs are not digested by humans and thus reach the human gut microbiota (HGM) intact, and through a huge repertoire of bacterial carbohydrate-active enzymes can be digested [3, 7]. Currently, what happens between the microorganisms and the host after EMP ingestion is a hot topic of research. However, most of the studies are conducted employing single bacterial strains, without considering the microbial networking established in the HGM [8]. Bacillota and Bacteroidota (formerly Firmicutes and Bacteroidetes, respectively [9]) represent around 90% of the total bacterial abundance [8, 10, 11]. Other phyla are Pseudomonadota (Proteobacteria), Actinomycetota (Actinobacteria), and Verrucomicrobiota (Verrucomicrobia) [9, 12]. Within the ecosystem, it is possible to decipher keystone bacteria that are essential to keep biodiversity high even if they have relatively low abundance [13, 14]. This concept is linked to the ability to support a cross-feeding mechanism within the community, in particular in presence of complex carbohydrates or substrates that can produce intermediates supporting the growth of other anaerobic intestinal bacteria. For example, *Akkermansia muciniphila* (the only Verrucomicrobiota) degrades endogenous mucus, generating acetate and succinate, enabling other microorganisms to produce butyrate via acetyl-CoA pathway [7, 12]. Pseudomonadota consume oxygen, promoting the growth of beneficial strict anaerobes and competing with pathogens through nutrient deprivation and the release of antimicrobial molecules [12]. Therefore, the strictly anaerobic Clostridiales (Bacillota) can take advantage, and then produce butyrate and secondary bile acids [12]. The facultative anaerobic Lactobacillales (Bacillota) release antimicrobial molecules, and metabolize amino acids into bioactive compounds, as well as releasing short-chain fatty acids (SCFAs) after fiber fermentation [12]. Moreover, Bifidobacteria (Actinomycetota) release simple sugars in the surrounding environment through monosaccharide-specific ATP-binding cassette transporters [12] that can be used by other bacteria.

One of the most important phyla, recognized as the HGM foundation taxon [13], is Bacteroidota whose members can break inaccessible dietary plant-derived carbohydrates, mucin-associated glycans, and host derived-polysaccharides [11] and make them more available to the intestinal community thanks to the highest range of polysaccharide utilization loci (PUL) in their genomes [12]. Altogether, these literature data reports that Bacteroidota, Bifidobacteria, and Bacillota organize the trophic chain of the gut ecosystem, supporting the growth of other anaerobic intestinal symbiotic bacteria [3, 7].

Thus, the established interactions between gut bacteria and the carbon source utilization are fundamental in maintaining intestinal homeostasis and beneficial effects mainly due to the metabolic end-products such as SCFAs which can derive from complex carbohydrates metabolism including polysaccharides of Maitake extract [15–18]. In fact, gut bacteria strengthen the intestinal barrier, boost the production of antimicrobial peptides, and reduce inflammation in the presence of SCFAs, enhancing mucosal homeostasis [3, 19, 20]. SCFAs have also a pleiotropic effect influencing distant body compartments via passive diffusion or by utilizing specific transporters [21, 22]. Through the gut-brain axis, the metabolites can even reach and cross the blood-brain barrier, influencing the release of neurotrophic factors [18]. Several cell types are responsive to SCFAs via G-protein-coupled receptors (GPCRs) [18, 22], such as GPR41 and GPR43. These are the major SCFA receptors regulating gene expression in immune cells, modulating the inflammatory cascades of nuclear factor kappa-light-chain-enhancer of activated B cells (NF-kB), extracellular signal-regulated kinases (ERK), and p38 mitogen-activated protein (MAPK). At the mucosal level, SCFAs promote the expansion of regulatory T cells (Tregs) and the release of interleukin-10 (IL-10), essential for limiting the pro-inflammatory response [22–24].

However, the effects of Maitake EMPs as prebiotic sources on the maintenance of intestinal homeostasis are still unclear. In this scenario, the administration of the selected prebiotic, and/or the suitable probiotic bacteria, or customized microbiome-based therapeutics seems to be a valuable approach to restore the “food-microorganism-SCFAs” axis impacting on the general health state [25, 26]. To establish a cause-and-effect relationship between treatments and outcomes, *in vitro* reconstructed HGM interfaced with *in vitro* models of the host is a promising strategy [27]. The available HGM models range from deep-well plates to super-controlled bioreactors, working in batch, semi-continuous, and continuous modes, as single or multi-stages. Their reliability is strongly impacted by the viability and fitness of the inoculated bacteria. The fresh gut microbiota is the gold standard, but the most difficult to handle. Nevertheless, metagenomic data show that metabolic pathways are stable among different microbiota, despite variations in community structure [10]. Therefore, microbiome engineers started to construct synthetic bacterial communities designed from a bottom-up (from the components to the communities) or a top-down (starting from the system) ecological perspective, taking advantage of descriptive mathematical models [28, 29]. Shetty et al. [30] designed a synthetic Diet-based Minimal Microbiome (Db-MM) of ten core intestinal bacterial species to study the efficiency of converting known dietary fibers into SCFAs. Medina et al. [31] focused their attention on the infant gut microbiome assembly, considering four representatives (*Bifidobacterium longum* subsp. *infantis*, *Bacteroides vulgatus*, *Escherichia coli*, and *Lactobacillus acidophilus*) and two different human milk oligosaccharides (fructooligosaccharides or 2-fucosyllactose), highlighting the establishment of cross-feeding interactions, sustaining the positive *B*. *infantis* growth. Thomson et al. [8] selected prominent adult gut bacteria (*Bifidobacterium adolescentis*, *Bacteroides dorei*, *E*. *coli*, *L*. *plantarum*, and *Clostridium symbiosum*), reporting that the supernatant derived from *B*. *dorei* e *C*. *symbiosum* on xylan resulted enriched in butyrate, reducing the inflammation of HT-29 cell line. The latest frontier of biomedical research has the ambitious project of translating this knowledge into beneficial medical therapies [12]. The goal of this research is to create rationally designed microbiome-based live biotherapeutics to complement missing or underrepresented functions in a dysbiotic microbiome, such as the one of IBD patients, addressing specific immunological targets [32].

The aim of the present work is the evaluation of the effects of a Maitake extract enriched in EMPs as a prebiotic [33] and a probiotic formulation on the modulation of a new *in vitro* reconstructed synthetic gut microbiota. The derived metabolites are administered to human immune and epithelial cells to have a comprehensive vision of what can happen in the mucosal intestinal environment.

## 2. Materials and methods

### 2.1 Extraction and characterization of Maitake mushroom bioactive molecules

**Fig 1:**
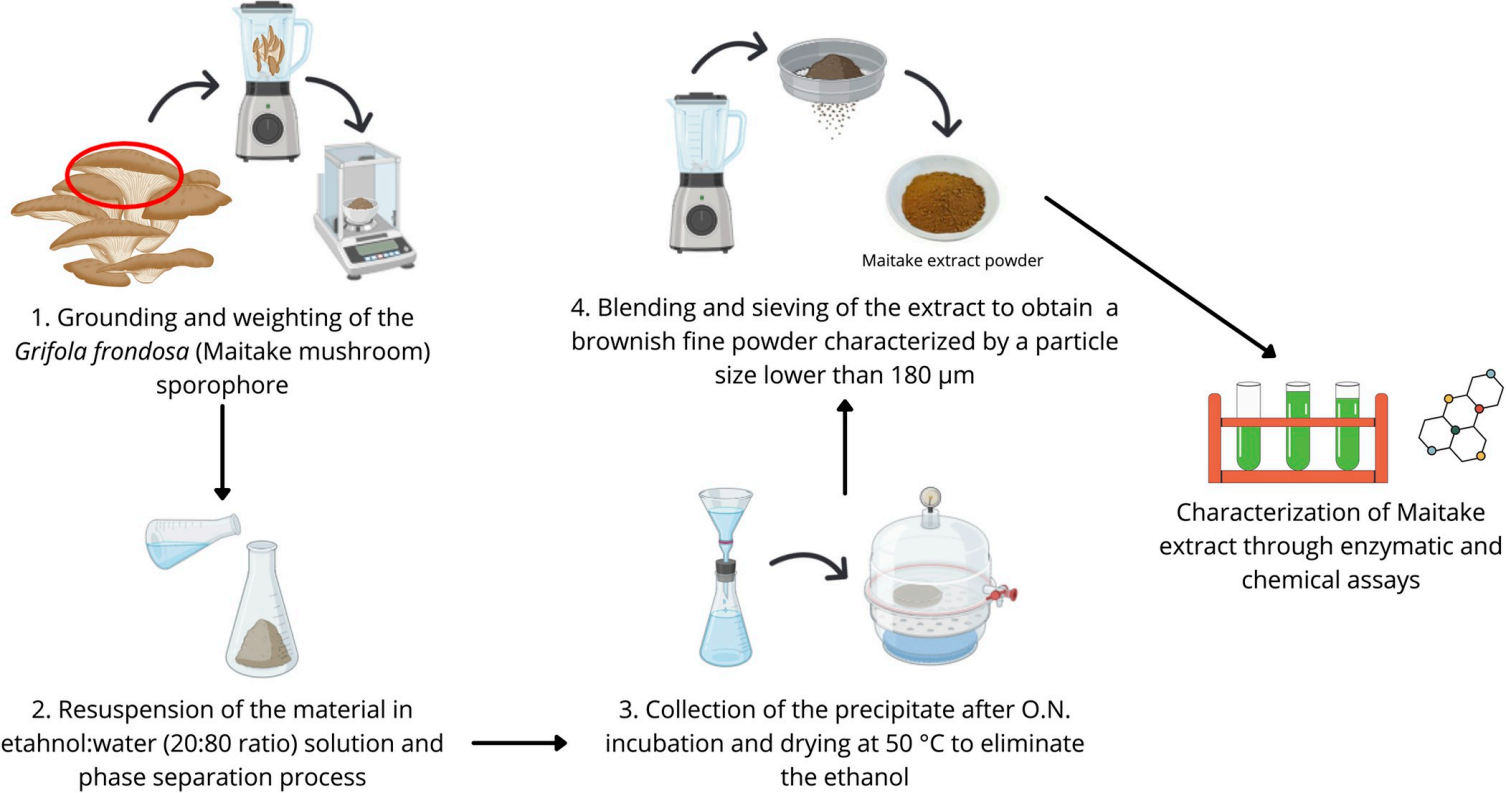
Schematic representation of the extraction method for polysaccharides from the fungus *Grifola frondosa* (Maitake). The image was created using Canva and Biorender.

The method for obtaining an extract enriched in polysaccharides from the sporophore of *Grifola frondosa* (Dicks) Gray, namely Maitake (Amita HC Italia S.r.l. Milan, Italy), is described in De Giani et al. [33] and shown in Fig 1. The maitake extract was obtained from an ethanol-water extraction and the material was dried resulting in a brownish fine powder (particle size < 180 μm).

The characterization of the principal components of the Maitake powder was determined through enzymatic commercial kits Megazyme (Megazyme Inc., Chicago, IL, USA). The components are reported in Table 1.

**Table 1:** Characterization of Maitake mushroom extract.

Component	Maitake extract (%)
**Starch**	49.5
**Total glucans**	25.0
**α-glucans**	6.2
**β-glucans**	18.8
**Proteins**	0.02
**Polyphenols**	1.9
**Fructans**	1.2
**Reducing sugars**	3.6

### 2.2 Bacterial strains and culture conditions

The bacterial strains used in this study are reported in a previous work [34] and listed in Table 2. The probiotics were kindly supplied by Roelmi HPC (Origgio, Italy) [35], while the minimal core microbiota strains belong to BEI Resources, NIAID, NIH collection, as part of the Human Microbiome Project. *Escherichia coli* ATCC 25922 is from the American Type Culture Collection (ATCC, Manassas, VA, USA).

**Table 2:** List of the bacterial strains used in the study.

Strain	Source	Abbreviation
*Lactobacillus acidophilus* PBS066 (DSM 24936)	Human	LA
*Limosilactobacillus fermentum* PBS073 (DSM 25176)	Human	LF
*Lactiplantibacillus plantarum* PBS067 (DSM 24937)	Human	LP
*Limosilactobacillus reuteri* PBS072 (DSM 25175)	Human	LR
*Lacticaseibacillus rhamnosus* LRH020 (DSM 25568)	Human	LRh
*Bifidobacterium animalis* subsp. *lactis* BL050 (DSM 25566)	Human	BL
*Bifidobacterium longum* subsp. *longum* BLG240 (LMG P-29511)	Human	BLg
*Bifidobacterium longum* subsp. *infantis* BI221 (LMG P-29639)	Human	BI
*Bacteroides cellulosilyticus* CL02T12C19, HM-726	Human	BC
*Bacteroides finegoldii* CL09T03C10	Human	BF
*Clostridium symbiosum* WAL-14673, HM-319	Human	CS
*Flavonifractor plautii* (formerly *Clostridium orbiscindens* 1_3_50AFAA, HM-303)	Human	FP
*Ruminococcus gnavus* CC55_001C	Human	RG
*Escherichia coli* ATCC 25922	Human	EC

The routine growth conditions consisted of De Man, Rogosa, and Sharp medium (MRS) (Conda Lab, Madrid, Spain) supplemented with 0.03% L-cysteine (Merck, Milano, Italy) for *Lactobacillus* and *Bifidobacterium* strains maintenance [34]; Reinforced Clostridia Medium (RCM) (Conda Lab, Madrid, Spain), supplemented with 0.03% L-cysteine, and 0.01 g/L of hemin (Hemin chloride, Cayman Chemical, Ann Arbor, MI, USA) for the minimal core microbiota [14].

The growth experiments with single strains were conducted in 24 multiwells (24 wells, SPL Lifesciences, Pocheonsi, Korea) in a final volume of 1 mL. Maitake power was added to a modified MRS (mMRS) [36] to a final concentration of 2% *w/v* and then together sterilized before inoculation. The initial Optical Density at 600 nm (OD_600nm_) of each single strain was 0.1. All the strains were grown statically in anaerobiosis (Anaerocult GasPack System, Merck, Darmstadt, Germany) at 37°C for at least 48 hours and the OD_600nm_ was measured.

### 2.3 Experiments with minimal core microbiota and probiotics in batch fermentation system

The minimal core microbiota strains were pre-inoculated for 72 hours, *L*. *plantarum* PBS067 (LP), *L*. *acidophilus* PBS066 (LA), and *B*. *animalis* subsp. *lactis* BL050 (BL) for 48 hours as described in 2.2 section at 37°C in anaerobic condition before the set-up of the experiments.

The three, four, or seven-species consortia were cultured in a 400 mL batch reactor (Colaver, Vimodrone, Italy), using mMRS (negative control), or mMRS supplemented with 2% Maitake or 2% fructooligosaccharides (FOS, 3 < DP < 5, Farcoderma, Torre Pallavicina, Italy; positive control) as carbon sources. In all the experimental conditions, the batch reactor was stirred at 60 rpm and maintained under anaerobic conditions by blowing nitrogen 5.0 (Sapio, Monza, Italy) for 48 hours at 37°C. Under sterile conditions, the medium was inoculated with a proper volume of each bacterial strain to have an initial OD_600nm_ of 0.05 as suggested by Isenring et al. [10].

To follow the different experimental sets over time, sampling for successive analysis was done by taking manually 10 mL from the batch reactor every 8 hours (T0, T8, T16, T24, T32, and T48). Before and after each sampling, nitrogen was flushed for 10 min to remove the possible oxygen entering. Then the OD_600nm_ was registered. Samples were centrifuge (Eppendorf, Milano, Italy) at 7,000 rpm for 10 min. Supernatants were analyzed for microbial metabolites, including SCFAs and branched-chain fatty acids (BCFAs) production as described in 2.6 section. Bacterial cells were stored for DNA extraction and bacterial species quantification by qPCR assays as described in 2.4 and 2.5 sections, respectively.

### 2.4 Total DNA extraction from single strains and *in vitro* reconstructed HGM in batch fermentation

Total DNA was extracted from single bacterial cultures (title of around 10^8^ CFU/mL) for the construction of standard curve to quantify the strains by qPCR analyses [37, 38]. Total DNA was obtained by Ultraclean Microbial DNA Isolation Kit (Qiagen, Milano, Italy). For the minimal core microbiota DNA extraction, a heat break step was included as indicated in the protocol.

The total DNA of the three tested conditions in batch fermentations was extracted using Stool Nucleic Acid Isolation Kit (Norgen Biotek Corp., Thorold, Canada) following the protocol provided by the manufacturer with some modifications [38].

DNA concentrations and purity were evaluated spectrophotometrically (NanoDrop One Microvolume UV-Vis Spectrophotometer, ThermoFisher Scientific, Monza, Italy).

### 2.5 Monitoring of the modulation of the strain abundances of the *in vitro* reconstructed HGM through qPCR

qPCR reactions were conducted using PCR Real-Time StepOne Plus (Applied Biosystems, Monza, Italy) and the PowerUp SYBR Green Master Mix (Applied Biosystems, Monza, Italy). Some species-specific primer sets utilized in this study were developed in a previous work by the authors [37], while others were designed specifically for the experiments (S1 Table) [39–42]. PCR reactions were carried out in a 10 μL qPCR mix containing the PowerUp SYBR Green Master Mix, Forward and Reverse primers (10 μM each), and 20 ng/μL of DNA template. Different qPCR programs were employed. For DNA amplification of LP, LA, BL, BC, CO, and EC strains the program was of 40 cycles of 15 seconds at 95°C and 1 minute at 60°C [38]; for CS, 40 cycles of 5 seconds at 95°C, and 30 seconds at 60°C (adapted from Ogita et al. [43]). Each DNA sample was analyzed in triplicate.

### 2.6 Extraction and characterization of microbial metabolites derived from Maitake or FOS fermentation

The extraction of bacterial metabolites was performed after acidification (until pH 2 with 6 M HCl) of the collected cultural broths. Ethyl acetate (Merck, Milano, Italy) was used as a solvent for the extraction in a 1:1 ratio to the acidic broth culture, and the extraction was conducted as reported in De Giani et al. [34].

A gas chromatographic instrument (Technologies 6890 N Network GC System, Agilent Technologies, Santa Clara, CA, USA) equipped with a mass selective detector (5973 Network, Agilent Technologies, Santa Clara, CA, USA) was employed for the metabolite analysis. Analyses were carried out in splitless injection mode in a capillary column (J&W DB-5ms Ultra Inert GC Column, fused silica, 60 m × 0.25 mm, 0.25 μm, Agilent Technologies, Santa Clara, CA, USA) with 99.99% He as carrier gas (Sapio, Bergamo, Italy). The hoven setting was 65°C for 2 min, followed by 8°C/min to 110°C, then 17°C/min to 260°C, holding the temperature for 10 min. Specific masses (73, 75, 117, 129, 132, 145, 159, 171, 173, 187, 201, 215, 229, 243, and 257 *m/z*) at 70 eV were selected for the analyses. All the samples were injected three times. The obtained chromatograms and mass spectra were interpreted by comparison with the one present in the National Institute of Standards and Technology (NIST), and with injected standard molecules (Merck, Milano, Italy).

### 2.7 *In vitro* experiments on human cell lines

#### 2.7.1. Participants as healthy donors to the Ethic Committee

Healthy donors’ peripheral blood buffy coats were obtained from the European Institute of Oncology (IEO Hospital), Milano, Italy. Written informed consent was obtained as standard practice from the donors at the IEO to donate the buffy coats for research purposes. The Institutional Review Board (Ethic Committee of the Hospital) approved the study N. DA-IEO 566. All methodologies were in full compliance with the Declaration of Helsinki.

#### 2.7.2. Human cell lines

Peripheral blood mononuclear cells (PBMCs) were isolated by density gradient centrifugation using Ficoll-Paque as standard protocol and maintained in culture in RPMI (Gibco, Monza, Italy).

The human intestinal epithelial HT-29 cell line (ATCC, Manassas, VA, USA) was cultured in DMEM (Gibco, Monza, Italy). Both media were supplemented with 10% FBS (Sigma-Aldrich, Milano, Italy) and 1% of Penicillin and Streptamycin (Gibco, Monza, Italy). Cells were maintained in an atmosphere containing 5% CO_2_ at 37°C.

PBMCs and HT-29 cells were cultured in the presence of the obtained bacterial metabolites at a fixed concentration of 0.5 mg/mL for 48 hours, then supernatants were collected to evaluate cytokines production. PBMC phenotype and function were evaluated through flow cytometry, while total RNA was extracted from HT-29 cells.

### 2.8 Cytofluorimetric and cytokine analyses on PBMCs

PBMCs were stained with combinations of directly conjugated antibodies (as specified in S2 Table).

Intracellular cytokines were detected after stimulation for 3 hours with 50 ng/mL PMA and 1 μg/mL ionomycin in the presence of 10 μg/mL Brefeldin A (reagents from Sigma, Milano, Italy). Cells were fixed and permeabilized with FOXP3 Transcription Factor Staining Buffer Set (eBioscience, Milano, Italy).

Samples were analyzed with a FACSCelesta flow cytometer (BD, Milano, Italy), gated to exclude singlets based on light scatter, using FlowJo v10 software (BD, Milano, Italy). The laser wavelengths were Violet, Blue, and Red. The utilized filter configurations and the specific voltage settings were 405 nm for BV510 (490 V) and BV786 (634 V); 488 nm for FITC (480 V), PE (400 V), and PE-Cy7 (649 V); 635 nm for APC (574 V) and APC-Cy7 (466 V). The acquisition speed was 50–100 events per second.

Cytokines (IL-6, IL-8, and IL-10) were measured by ELISA (Ab are reported in S3 Table) performed following the manufacturer’s instructions.

### 2.9 Total RNA analyses from epithelial cells

Total RNA from HT-29 cells was isolated using TRIzol (Invitrogen, Monza, Italy) and Quick-RNA MiniPrep (ZymoResearch, Freiburg im Breisgau, Germany) according to the manufacturer’s specifications. cDNAs were generated from 1 μg of total RNA with EasyScript Plus cDNA Synthesis kit (abm, Roma, Italy). Gene expression levels were evaluated by qPCR and normalized to RPL32 gene expression. Human primers are listed in S4 Table.

### 2.10 Statistical analyses

Regarding the bacterial growths, the statistical relevance of the results was assessed by the t-Student test. The significance was defined as * *p*-value < 0.05 or ** *p*-value < 0.01. The prebiotic index (PI) was calculated as reported by Palframan et al. [44].

The resulting data on the quantification of bacteria by real time q-PCR were expressed as mean values ± standard error (SE). The average slope and y-intercept of each standard curve were determined by regression analyses and used to calculate the bacterial counts/mL for each bacterial target.

Concerning the experiment with immune and epithelial cell lines, the statistical relevance of the data was assessed by ANOVA. The significance was defined as * *p-*value < 0.05, ** < 0.01, or *** < 0.001.

## 3. Results

### 3.1 Single strain growths on prebiotic Maitake extract

**Fig 2:**
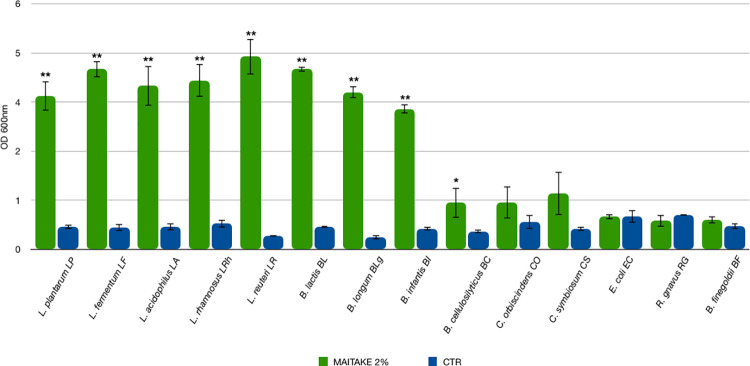
Growth levels of individual bacterial strains on Maitake extract. The growth is presented as the mean value of OD_600nm_ ± SE in the presence of 2% Maitake extract and the CTR medium. Statistical differences were calculated using t-Student’s test: * *p—*value <0.05, ** < 0.01.

The possible capability of Maitake extract to sustain the growth of several bacterial strains composing the gut microbiota at a final concentration of 2% *w/v* was assessed. As shown in Fig 2, all the probiotics could utilize Maitake to grow, reaching an OD_600nm_ value higher than 4.0 (*p-*value < 0.01). The minimal core microbiota strains arrived at a final OD_600nm_ of 1.0. The calculated prebiotic index PI, considering the growth of Bifidobacteria (Bif), Bacteroides (Bac), Lactobacilli (Lac), and Clostridia (Clos), showed a value of 2.06, while the CTR PI value was around 0 (-0.18).

Accordingly with these results, the Maitake extract at 2% concentration was selected as prebiotic for the next experiments.

### 3.2 Validation of the *in vitro* reconstructed HGM

To understand whether the selected strains (Table 2) composing the *in vitro* reconstructed HGM worked, we conducted preliminary experiments using FOS as prebiotic source. Results indicated that the highest OD_600nm_ equal to 3.48 ± 0.26 is achieved by the probiotics after 32 hours of fermentation, while the lowest (OD_600nm_ of 2.45 ± 0.25) is of the minimal core. The growth peak of the full community is reached at 24 hours and is equal to OD_600nm_ 2.94 ± 0.19 (S1A Fig). Looking at single strain amounts, in the condition with only probiotics, LP reaches a value in the order of 10^9^ bacterial counts/mL at 32 hours (*p-*value < 0.05 vs time 0). In the only minimal core set-up, BC reaches the highest bacterial counts/mL value (on the order of 10^8^) at 24 hours (*p-value* < 0.05 vs time 0). Considering the total community, the strains that are favored are LP and BC, which arrive at bacterial counts/mL values in the order of 10^9^ (*p-*value < 0.05 vs time 0) and BL reaches bacterial counts/mL values in the order of 10^10^ (*p-*value < 0.05 vs time 0) (S1B Fig).

The analyses of released bacterial metabolites reveal lactic, butyric, valeric, and propionic acids in the three different set-ups, which belong to the bacterial fermentation of a prebiotic substrate (S1C Fig).

### 3.3 *In vitro* reconstructed HGM modulation by Maitake extract

**Fig 3:**
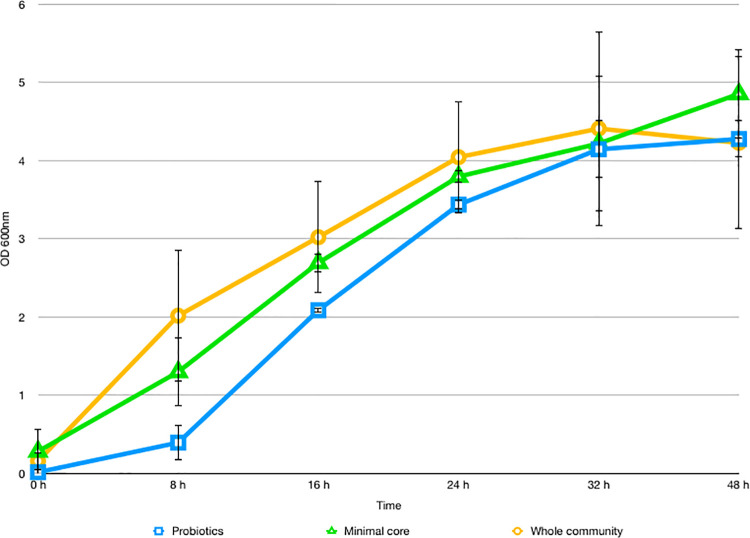
Growth curves of probiotics, minimal core, and the whole community in batch fermentation. The figure depicts the growth curve (mean value of OD_600nm_ ± SE during time) of only the probiotics, only the minimal core strains and the whole *in vitro* gut microbiota in the presence of 2% Maitake extract in batch fermentation.

**Fig 4:**
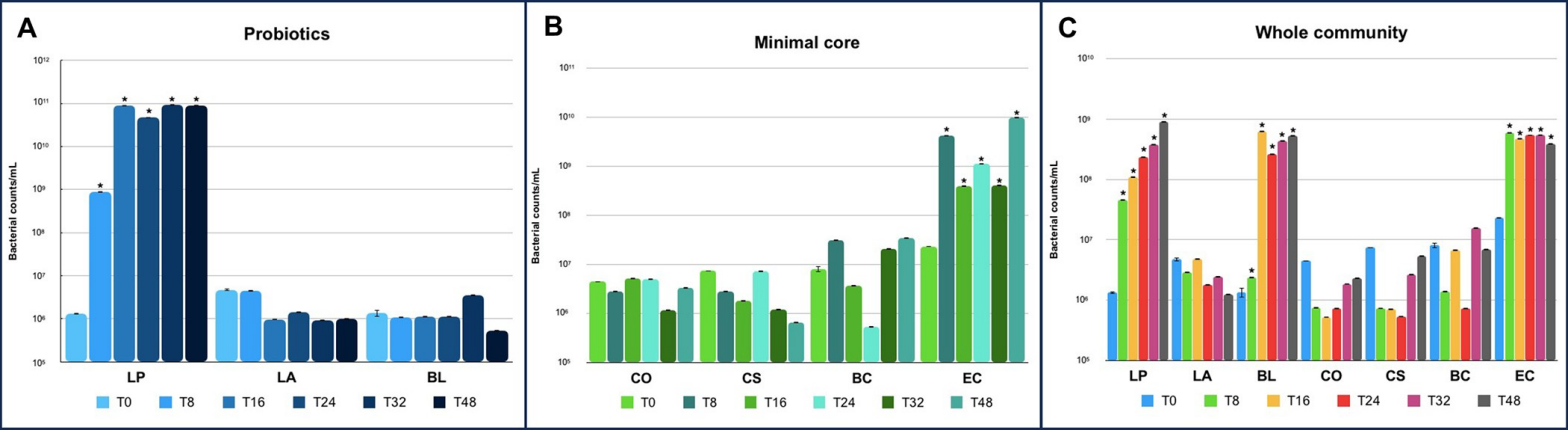
Single strain level modulation. The figure represents the bacterial counts/mL of, the probiotics (A), the minimal core strains (B), and the whole community (C) obtained after qPCR analyses with species-specific primers during the growth on 2% Maitake extract. Statistical differences were calculated using t-Student’s test: * *p—*value < 0.05.

Based on the validation of the *in vitro* HGM reconstruction results, we tested the defined microbiota configuration in the presence of Maitake as prebiotic source at 2% concentration. As shown in Fig 3, the minimal core microbiota reached an OD_600nm_ of 3.57 ± 0.07 after 24 hours. The probiotic bacteria alone showed the highest OD_600nm_ after 32 hours (3.88 ± 0.33). The growth of the whole microbial community reached the maximum after 32 hours (OD_600nm_ of 4.11 ± 1.09. By qPCR, results showed that, among the probiotics, LP prevails over other strains reaching a bacterial counts/mL value in the order of 10^11^ at 24 hours (*p-*value < 0.05 vs time 0) (Fig 4A). While, in the set-up with only the minimal core strains, EC reaches a maximum value in the order of 10^10^ bacterial counts/mL at 48 hours (*p-*value < 0.05 vs time 0) (Fig 4B). In the whole community batch, LP, BL, and EC reach values in the order of 10^8^−10^9^ bacterial counts/mL (*p-*value < 0.05 vs time 0) between 24 and 32 hours, but with very different growth trends (Fig 4C). Indeed, EC immediately reaches a plateau, while the growth of the probiotics is favored over time. The calculation of the PI during the time point, considering the specific bacterial counts/mL of each strain, reveals a value of 2.9 at the end of the growth, supporting the prebiotic action of the Maitake extract and the goodness of the *in vitro* HGM model.

### 3.4 Monitoring the production of microbial metabolites and SCFAs by the *in vitro* reconstructed HGM

**Fig 5:**
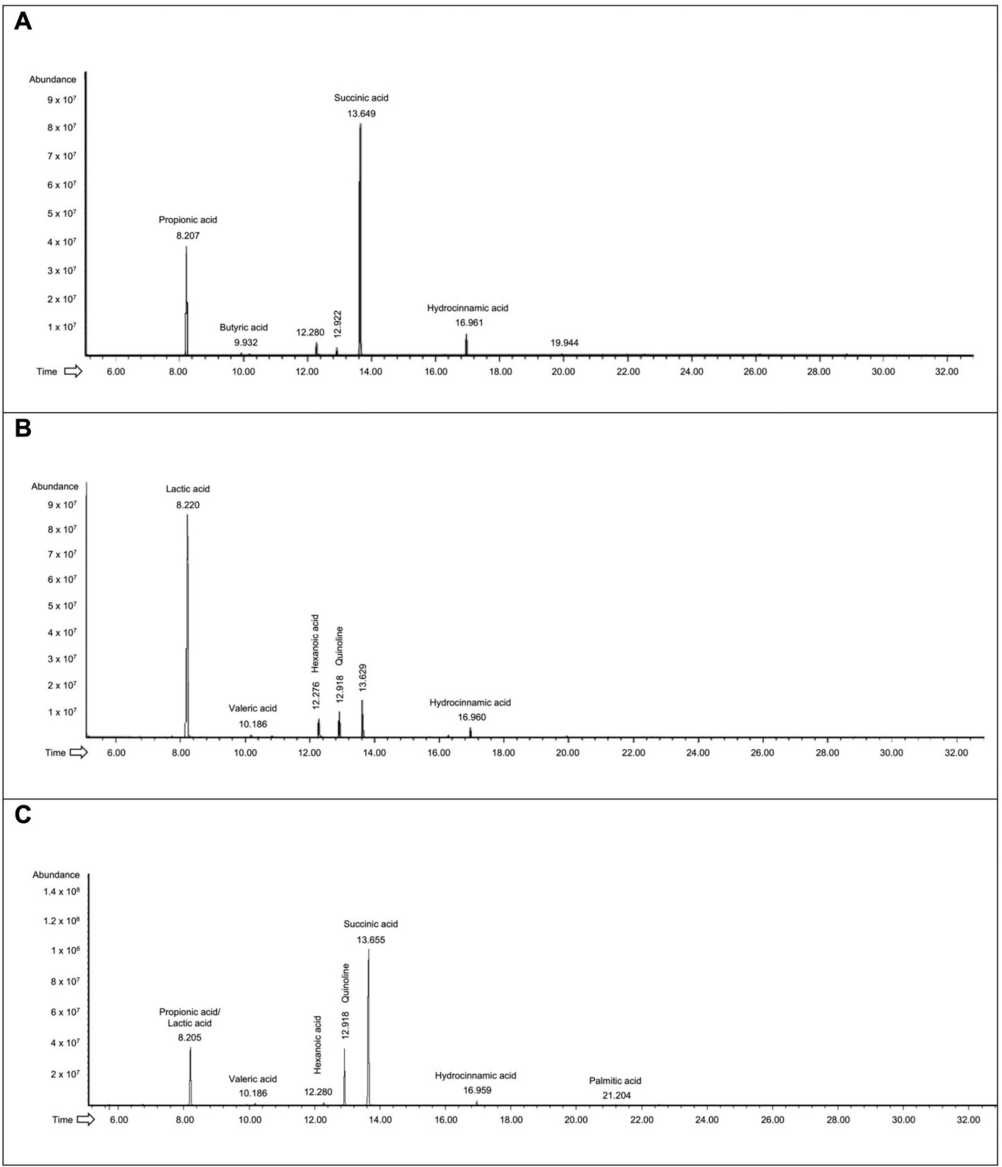
Analyses of bacterial metabolites by GC-MSD after Maitake extract fermentation. In (A) is reported the chromatogram regarding the metabolites produced by the minimal core strains, in (B) the metabolites released by the probiotics, and in (C) the chromatogram of the whole community.

The metabolites produced after Maitake fermentation in all the experimental sets were extracted and analysed. The chromatogram in Fig 5A regards the condition with only the minimal core strains, at the final time point (T48). The first peak was at a retention time (R_t_) of 8.2 min, and it was assigned to propionic acid. The second important peak was at an R_t_ of 13.65 min, corresponding to succinic acid. Other detected molecules were butyric (R_t_ of 9.93 min) and hydrocinnamic acid (R_t_ of 16.96 min). Other compounds were revealed, but it was not possible to assign them to the corresponding molecules. The chromatographic profile was consistent during all the considered time points.

Considering the Maitake fermentation by the probiotic strains at 48 hours (Fig 5B), the first important peak at R_t_ of 8.2 min was associated with lactic acid. The peak at 12.2 min was hexanoic acid, and the third highest peak was quinoline (R_t_ of 12.9 min). Other molecules were valeric acid (R_t_ of 10.18 min) and hydrocinnamic acid (R_t_ of 16.9 min). Also in this case, the molecules could be detected all over the considered time points.

Finally, the whole community condition was analyzed at the final time point (Fig 5C). The first detected molecule was at R_t_ of 8.20 min, which could be assigned to lactic or propionic acid (or a mixture of both), which are both SCFAs. Then, at R_t_ of 10.18 min, there is valeric acid, at 12.28 min hexanoic acid, and another high peak corresponding to succinic acid (R_t_ of 13.66 min). Both quinoline (12.9 min R_t_) and hydrocinnamic acid (16.95 min R_t_), corresponding to mushroom polyphenols, were detected. Finally, after 21.2 min, also palmitic acid was identified.

### 3.5 Functional effects of bacterial metabolites and SCFAs on epithelial cells and immune cells

**Fig 6:**
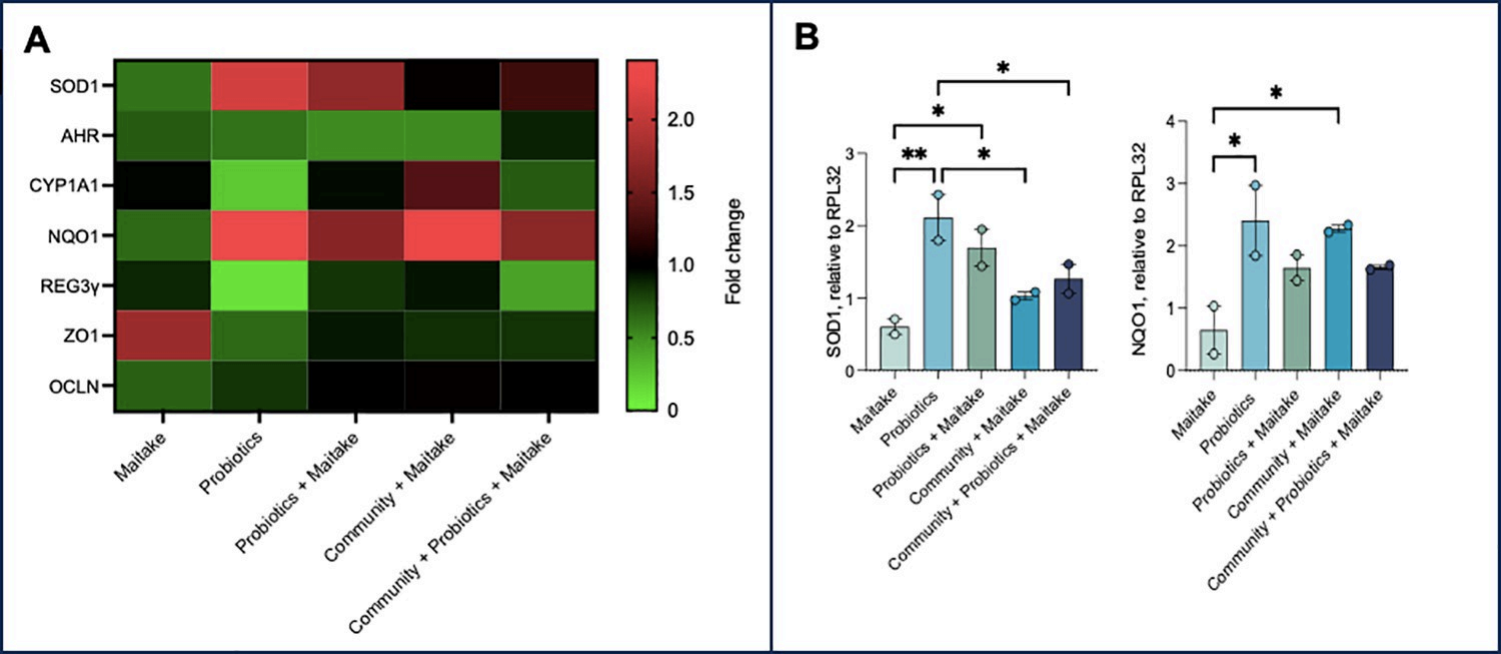
Antioxidant response of HT-29 cell line. After the treatment of the cells with a fixed concentration of the different metabolites and the Maitake extract alone for 48 hours, the expression of specific genes was verified via qPCR analyses. The heatmap (A) reports the modulation considering the fold change with respect to a reference gene. In (B) is highlighted the increment of SOD1 and NQO1 genes with respect to the RPL32 gene as mean value ± SD. The statistical differences are calculated by parametric ANOVA test.

The bacterial metabolites were administered to human intestinal epithelial cell lines for 48 hours and the relative expression of selected genes was evaluated. Interestingly, our results suggest a modulation of genes involved in oxidative stress pathways given by the different bacterial communities. In particular, our results showed increased expression of superoxide dismutase 1 (SOD1) (*p-*value < 0.05) and NAD(P)H-quinone-dehydrogenase 1 (NQO1) (*p-*value < 0.05) in cells treated with metabolites resulting from the fermentation of Maitake by probiotics and minimal core microbiota, respectively, but not in the presence of Maitake extract alone (Fig 6A and 6B). The effect is due to the presence of these specific metabolites because the molecules released after FOS fermentation do not mediate this kind of response, even if common SCFAs are present (S1 and S2 Figs).

**Fig 7:**
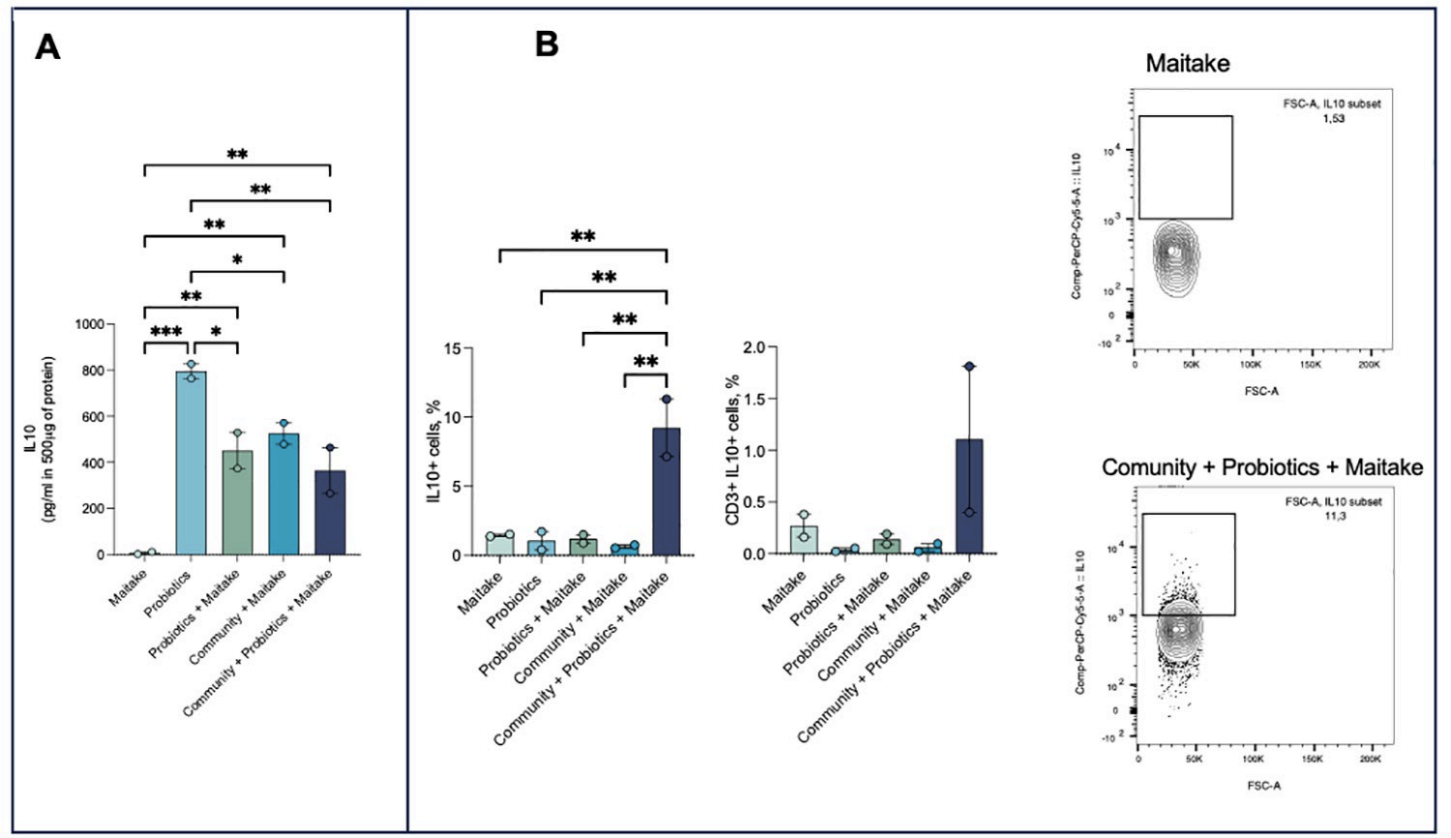
Anti-inflammatory response of PBMCs. The production of anti-inflammatory IL-10 was evaluated through ELISA tests (A) in the presence of a fixed concentration of the different bacterial metabolites or Maitake extract alone. Quantities are expressed as mean values ± SD. The statistical differences were calculated by ANOVA. In (B) are reported the results of the FACS analyses as cells producing IL-10 with respect to FSC-A.

At the same time, we exposed the metabolites from Maitake extract fermentation to freshly isolated human PBMCs [45, 46] for 48 hours at a fixed concentration. The stimulation of PBMCs with bacterial metabolites induced these cells to produce significantly higher levels of IL-10 compared with cells treated with Maitake extract alone (metabolites from the whole community *vs* Maitake: *p-*value < 0.01) (Fig 7A). No differences were observed in the production of the pro-inflammatory cytokines IL-8 and IL-6 (data not shown). To confirm these data, we performed a flow cytometry analysis. The results showed that not only T cells but all the immunological compartment was skewed toward IL-10 production, regarding the condition in which PBMCs were treated with metabolites derived from the fermentation of Maitake by the whole community (*p-*value < 0.01) (Fig 7B). Interestingly, the PBMCs treated with the metabolites deriving from the fermentation of the control molecules FOS have a much lower anti-inflammatory power than the treatment with metabolites from the fermentation of Maitake. Furthermore, the percentage of cells producing IL-10 did not change between control and treatment (S2 Fig).

All together, these results suggest a potential role played by the synergic activities of Maitake, probiotics, and the microbial community in inducing a tolerogenic microenvironment.

## 4. Discussion

In the present work, a new *in vitro* HGM resembling the adult microbe-microbe networking was reconstructed to better comprehend the complexity of the human intestinal ecosystem and its relevance for human health. The *in vitro* HGM was conceived considering *E*. *coli*, *B*. *cellulosilyticus*, *F*. *plautii*, and *C*. *symbiosum* as members of the minimal core (Table 2) because they belong to Pseudomonadota, Bacteroidota, and Bacillota phyla, respectively. The selected set-up embraces what is currently known about the composition of the gut microbiota *in vivo* thanks to the numerous published papers regarding clinical trials and reconstructed gut communities [8, 27, 38]. Both Eckburg et al. [47] and Vaga et al. [48] analyzed mucosal and fecal samples obtained from healthy subjects. They showed that most of the microorganisms belong to the Bacillota and Bacteroidota phyla. Among the Bacillota, 95% of the sequences matched with Clostridia, and many are butyrate-producing bacteria. Among the Bacteroidota, the Bacteroides species are more abundant in association with the mucosa with respect to the feces. Relatively low sequences belong to Pseudomonadota, Actinomycetota, and Verrucomicrobiota. Therefore, they propounded a crucial role of Bacteroidota, while Bacillota and other dominant phyla play interchangeable functions. Concerning Actinomycetota in our *in vitro* HGM configuration, we thought of a probiotic intervention to restore a possible gut dysbiosis, supplying *B*. *animalis* subsp. *lactis* as representative of the Actinomycetota phylum as well as *L*. *plantarum L*. *acidophilus* (Table 2). They have an important interconnection degree with the other members of the intestinal community, positively correlating with diversity both within and between individuals [13].

The *in vitro* HMG set-up of this paper is inspired by the outcomes of a previous clinical study conducted by our group, in which the three selected probiotic strains combined with the same FOS were administered to healthy elderly subjects for 28 days [38]. In this case, we observed that the *Bacteroides* subsp. were the most modulated microorganisms by the administration of probiotics plus prebiotics. Moreover, *L*. *plantarum* and *B*. *animalis* subsp. *lactis* showed a similar increasing trend both *in vivo* and *in vitro*, data in line with the bacterial cell densities indicative of the model quality described in Isenring et al. [10]. Based on the valuable outcomes, we decided to challenge our *in vitro* HGM with a different candidate, Maitake extract, as a prebiotic which has never been in depth investigated in this kind of reconstructed model. The novelty of the paper is to consider complex carbohydrates such as those of Maitake extract to better understand the interactions between gut bacteria and the carbon source utilization, also considering their metabolic products to support a cross-feeding mechanism within the synthetic community in maintaining intestinal homeostasis.

First, the utilization of Maitake at 2% concentration as prebiotic source by the three probiotics and the four minimal core strains individually cultivated was evaluated. The data confirmed the prebiotic potential. Moreover, the computed prebiotic index [44] at pH 6.8 has a value around 2 for Maitake, which is intermediate between isomalto-oligosaccharides, lactulose, and FOS having values of 3.91, 3.00, and 0.77, respectively [44]. The outcomes confirmed the results obtained in other studies that employed *L*. *acidophilus*, *Lacticaseibacillus casei*, and *B*. *animalis* subsp. *lactis* in the presence of Maitake [49, 50].

Our mushroom extract from *Grifola frondosa* is enriched in β-glucans (recognized as EMPs), known for their beneficial effects on the host [3]. Few studies report the production of butyrate after prebiotic Maitake utilization [51–53]. However, the precise mechanism of β-glucan metabolism by every commensal gut bacterium is not elucidated yet. Only the recent work of Fernandez-Julia et al. [54] presents a cross-feeding approach employing co-cultures of *Bacteroides cellulosilyticus*, *Bifidobacterium*, and *Lactiplantibacillus* in the presence of β-glucans. In this case, the authors suppose that the *Bacteroides* strain has two extracellular enzymes that can degrade the complex polysaccharides, and then release oligosaccharides in the medium. These can be utilized by the secondary degrader bacteria, such as *Lactiplantibacillus*, to grow. Thus, EMPs from mushrooms can modify gut microbiota composition and function in a positive way [55].

In this scenario, we employed an *in vitro* HGM to investigate the possible interactions established within the various microbial communities in the presence of Maitake extract.

In particular, we observed that the exponential growth phase of the three chosen conditions (only probiotics, only the minimal core microbiota, and both) started from 8 hours of incubation, while the onset of the stationary phases was around 32 hours, except for the minimal core strains. An onset of growth slowdown is observable after 24 hours, as expected. The obtained results are in line with the literature. Indeed, it is known that *Bifidobacterium* and *Bacteroides* genera are primary degraders in the gut ecosystem [56] breaking down the complex polysaccharides into smaller ones and releasing metabolites such as acetate and lactate [8, 56]. In our experiments, BL has a very low abundance, and lactic acid was detected in the probiotic consortium alone, while BC has about the same abundance both in the presence of only minimal core bacteria and in the total community. However, acetate has not been detected, though it can be assumed that it may have been utilized by the other bacteria. Indeed, in the cross-feeding circle, acetate, and lactate are consumed to produce butyrate [8, 31], while the polysaccharides can be further fermented by bacteria present in smaller percentages, such as *E*. *coli* and *L*. *plantarum*. This is evident in all the tested conditions. *L*. *plantarum* is the most abundant bacterium in the only probiotic and the whole community experiments, while *E*. *coli* is dominant over BC, CS, and FP, and it is the second most present in the complete microbiota condition. Interestingly, *L*. *plantarum* biotransforms and grows on polyphenols present in natural extracts, releasing additional molecules [25, 33, 34], which in the presence of Maitake are quinoline [57] and hydrocinnamic acid, derived from cinnamic acid [58]. LP could be also correlated to lactic acid because it has an optional heterofermentative metabolism leading to the production of large quantities of the molecule in anaerobiosis [14]. Finally, the included Clostridia of the XIV and IV group produce butyrate from lactate [8]. *C*. *symbiosum* is known to be the most sensitive to the lack of primary degraders, though is stimulated by the presence of *E*. *coli*, *Bifidobacterium adolescentis*, *Bacteroides dorei*, and *L*. *plantarum* [14]. This effect is hardly visible in our experiments, even if the relative abundance is higher in the presence of EC and LP than in the presence of EC alone. Still, we detected butyrate, which can be produced by the bacterium starting from acetate and lactate, boosting the cross-feeding circle [14]. *F*. *plautii* (*C*. *orbiscindens*) always shows slight growth due to its special requirements, but it has an important role in community fitness [14]. Finally, the production of propionate in the presence of *B*. *cellulosilyticus* growth was expected and it could be also indirectly produced by EC and CS [14]. The established networking *in vitro* leads to different released metabolites, which are the main molecules interacting with the host and have been implicated in the maintenance of intestinal homeostasis. If perturbed, pathological conditions can be triggered [23]. The re-equilibration has been shown to contribute to the resolution of inflammation by acting on both immune and epithelial cells’ functions [59]. Butyrate can boost the tight junction protein complex integrity in the intestinal epithelium triggering the AMP-activated protein kinase (AMPK) signaling, increasing the expression of the tight junction (TJ) protein claudin-1, and inhibiting the claudin-2 TJ through IL-10-RA stimulation [60]. However, the created subtle balance can also be affected by the production of potentially harmful molecules from the host itself, such as reactive oxygen species (ROS), which play a pivotal role in cancer development and progression [61]. Hence, understanding the mechanisms behind their generation and counterbalance can be essential for intestinal health. SCFAs can mitigate stressful conditions through the stimulation of glutathione [60] and other scavengers. Superoxide dismutases (SODs) are enzymes that can reduce superoxide anions to hydrogen peroxide [62]. The cysteine transporter SLC7A11 and the quinone oxidoreductase (NQO1) are antioxidant genes induced by the transcription factor nuclear factor erythroid 2/related factor 2 (NRF2), which is sensible to the presence of butyrate and propionate [63]. The aryl hydrocarbon receptor (AHR) and its effector CYP1A1 also mediate oxidative stress using different mechanisms [64]. The prebiotic properties of Maitake extract on probiotic strains promote epithelial cell vitality recovery after oxidative stress injury. Moreover, we found that the cell line treated with bacterial-derived metabolites had increased expression levels of SOD1 and NQO1, suggesting that these pathways could be involved in cell recovery after oxidative stress.

Regarding the immunological compartment, the analysis of the response of PBMCs exposed to the extracted bacterial metabolites under the three tested *in vitro* HGM conditions showed that fermentation of Maitake extract by the complete *in vitro* microbiota (the closest simulation to the *in vivo* condition) induces a beneficial phenotype for the host, as the production of the anti-inflammatory cytokine IL-10 stimulated not only by T cells but by the whole immunological compartment. This response is not recorded in the presence of fungal extract alone or probiotic bacteria alone, to underlying that the beneficial effects shown are given precisely by the synergy established between bacteria components of the intestinal microbiota, probiotics, and Maitake extract. This emphasize that, though the gut microbiota plays a central role in host physiology and several pathological mechanisms, living microorganisms can be helpful as non-pharmacological methods to promote gut health since it is strongly involved in the functional plasticity of different immune cell types [45]. However, their beneficial effects are not merely dependent on the microorganisms but mostly on the metabolites they produce (i.e. SCFAs, bacteriocins, hydroperoxides, secondary bile acids, and lactic acids) [65]. Indeed, the presence of SCFAs producer bacteria in the gut microbiota induces an increase in IL-10 production and a tolerogenic phenotype in different immune cell populations [45, 59], as we have observed with our experiments.

## 5. Conclusions

In conclusion, this study demonstrates that the specific fermentation of Maitake extract by probiotic bacteria in the presence of a reconstructed minimal HGM mediates several positive effects. The analyses of the modulation of the gut bacterial community suggest how an intervention with pre- and probiotics could act in the adult gut. Furthermore, the Maitake raises the production of positive metabolites, that exert the effects on the host, both on the epithelium and the immune system. So, the results underly the important role of the administration of a probiotic treatment plus a suitable prebiotic in boosting a tolerogenic intestinal microenvironment. Furthermore, the work is placed in the two new potential horizons emerging in the biomedical field: the production and then administration of SCFAs or live microbial biotherapeutics to patients afflicted by inflammatory disorders [12, 66].

## Supporting information

S1 Fig Validation of the *in vitro* HGM on FOS. The growth is presented as OD_600nm_ (A) and bacterial counts/mL (B) during the time. The produced metabolites are illustrated as results of GC-MSD analyses (C).
(DOCX)

S2 Fig Antioxidant and anti-inflammatory response of cells exposed to FOS. In (A) are reported the fold change values of HT-29 *sod1* and *nqo1* with respect to the *rpl32* as mean values ± SD. The statistical differences are calculated by parametric ANOVA test. In (B) the production of anti-inflammatory IL-10 was evaluated through ELISA tests. Quantities are expressed as mean values ± SD. The statistical differences were calculated by ANOVA. The results of the FACS analyses as cells producing IL-10 are then reported.
(DOCX)

S1 Table List of the bacterial primer sets utilized in the study. (DOCX)

S2 Table Directly conjugated antibodies used for flow cytometry experiments. (DOCX)

S3 Table List of ELISA reagents. (DOCX)

S4 Table Primer list for human cell lines. (DOCX)

## References

[1] Li H, Tian Y, Menolli N, Ye L, Karunarathna SC, Perez-Moreno J. Reviewing the world’s edible mushroom species: A new evidence-based classification system. Food Sci Food Safety. 2021; 20: 1982–2014. doi: 10.1111/1541-4337.12708 . 33599116

[2] Maity P, Sen IK, Chakraborty I, Mondal S, Bar H, Bhanja SK, et al. Biologically active polysaccharide from edible mushrooms: a review. Int J Biol Macromol. 2021; 172: 408–17. doi: 10.1016/j.ijbiomac.2021.01.081 . 33465360

[3] Zhao Q, Jiang Y, Zhao Q, Patrick Manzi H, Su L, Liu D, et al. The benefits of edible mushroom polysaccharides for health and their influence on gut microbiota: a review. Front. Nutr. 2023; 10: 1213010. doi: 10.3389/fnut.2023.1213010 . 37485384 PMC10358859

[4] He X, Wang X, Fang J, Chang Y, Ning N, Guo H, et al. Polysaccharides in *Grifola frondosa* mushroom and their health promoting properties: A review. Int J Biol Macromol. 2017; 101: 910–921. doi: 10.1016/j.ijbiomac.2017.03.177 . 28366857

[5] von Martels JZH, Sadaghian Sadabad M, Bourgonje AR, Blokzijl T, Dijkstra G, Faber KN, et al. The role of gut microbiota in health and disease: In vitro modeling of host-microbe interactions at the aerobe-anaerobe interphase of the human gut. Anaerobe 2017; 44:3–12. doi: 10.1016/j.anaerobe.2017.01.001 . 28062270

[6] Rathore H, Prasad S, Sharma S. Mushroom nutraceuticals for improved nutrition and better human health: a review. PharmaNutrition 2017; 5: 35–46. doi: 10.1016/j.phanu.2017.02.001

[7] Chassard C, Lacroix C. Carbohydrates and the human gut microbiota. Curr Opin Clin Nutr Metab Care 2013; 16(4):453–60. doi: 10.1097/MCO.0b013e3283619e63 . 23719143

[8] Thomson P, Medina DA, Ortúzar V, Gotteland M, Garrido D. Anti-inflammatory effect of microbial consortia during the utilization of dietary polysaccharides. Food Res Int. 2018; 109:14–23. doi: 10.1016/j.foodres.2018.04.008 . 29803436

[9] Oren A, Garrity GM. Valid publication of the names of forty-two phyla of prokaryotes. Int. J Syst Evol Microbiol 2021; 71: 005056. doi: 10.1099/ijsem.0.005056 . 34694987

[10] Isenring J, Bircher L, Geirnaert A, Lacroix C. *In vitro* human gut microbiota fermentation models: opportunities, challenges, and pitfalls. Microbiome Res Rep. 2023; 2:2. doi: 10.20517/mrr.2022.15 38045607 PMC10688811

[11] McCallum G, Tropini C. The gut microbiota and its biogeography. Nat Rev Microbiol 2023. doi: 10.1038/s41579-023-00969-0 . 37740073

[12] Sorbara MT, Pamer EG. Microbiome-based therapeutics. Nat Rev Microbiol 2022; 20: 365–380. doi: 10.1038/s41579-021-00667-9 . 34992261

[13] Trosvik P, de Muinck EJ. Ecology of bacteria in the human gastrointestinal tract-identification of keystone and foundation taxa. Microbiome 2015; 3: 44. doi: 10.1186/s40168-015-0107-4 . 26455879 PMC4601151

[14] Gutiérrez N, Garrido D. Species deletions from microbiome consortia reveal key metabolic interactions between gut microbes. mSystems. 2019; 4(4):e00185–19. doi: 10.1128/mSystems.00185-19 . 31311843 PMC6635622

[15] Venturelli OS, Carr AC, Fisher G, Hsu RH, Lau R, Bowen BP, et al. Deciphering microbial interactions in synthetic human gut microbiome communities. Mol Syst Biol. 2018; 14(6): e8157. doi: 10.15252/msb.20178157 . 29930200 PMC6011841

[16] Faust K, Raes J. Microbial interactions: from networks to models. Nat. Rev. Microbiol. 2012; 10: 538–550. doi: 10.1038/nrmicro2832 . 22796884

[17] Pinto F, Medina DA, Perez-Correa JR, Garrido D. Modeling metabolic interactions in a consortium of the infant gut microbiome. Front Microbiol. 2017; 8: 2507. doi: 10.3389/fmicb.2017.02507 . 29312209 PMC5735223

[18] van der Hee B, Wells JM. Microbial Regulation of Host Physiology by Short-chain Fatty Acids. Trends Microbiol. 2021; 29(8): 700–712. doi: 10.1016/j.tim.2021.02.001 . 33674141

[19] Martin-Gallausiaux C, Marinelli L, Blottiere HM, Larraufie P, Lapaque N. SCFA: machanisms and functional importance in the gut. Proc. Nutr. Soc. 2021; 80(1):37–49. doi: 10.1017/S0029665120006916 . 32238208

[20] Hamer HM, Jonkers DM, Bast A, Vanhoutvin SA, Fischer MA, Kodde A, et al. Butyrate modulates oxidative stress in the colonic mucosa of healthy humans. Clin Nutr. 2009; 28(1):88–93. doi: 10.1016/j.clnu.2008.11.002 . 19108937

[21] Yao Y, Cai X, Fei W, Ye Y, Zhao M, Zheng C. The role of short-chain fatty acids in immunity, inflammation and metabolism. Crit Rev Food Sci Nutr. 2022; 62(1):1–12. doi: 10.1080/10408398.2020.1854675 . 33261516

[22] Luu M, Visekruna A. Short-chain fatty acids: Bacterial messengers modulating the immunometabolism of T cells. Eur. J. Immunol. 2019; 49: 842–848. doi: 10.1002/eji.201848009 . 31054154

[23] Perillo F, Amoroso C, Strati F, Giuffrè MR, Díaz-Basabe A, Lattanzi G, et al. Gut microbiota manipulation as a tool for colorectal cancer management: recent advances in its use for therapeutic purposes. Int J Mol Sci. 2020; 21(15):5389. doi: 10.3390/ijms21155389 . 32751239 PMC7432108

[24] Kaser A, Zeissig S, Blumberg RS. Inflammatory bowel disease. Annu Rev Immunol 2010; 28: 573–621. doi: 10.1146/annurev-immunol-030409-101225 . 20192811 PMC4620040

[25] De Giani A, Pagliari S, Zampolli J, Forcella M, Fusi P, Bruni I, et al. Characterization of the biological activities of a new polyphenol-rich extract from cinnamon bark on a probiotic consortium and its action after enzymatic and microbial fermentation on colorectal cell lines. Foods 2022a; 11:3202. doi: 10.3390/foods11203202 37430951 PMC9602362

[26] Shuwen H, Miao D, Quan Q, Wei W, Zhongshan Z, Chun Z, et al. Protective effect of the "food-microorganism-SCFAs" axis on colorectal cancer: from basic research to practical application. J Cancer Res Clin Oncol. 2019; 145(9):2169–97. doi: 10.1007/s00432-019-02997-x . 31401674 PMC11810311

[27] Elzinga J, van der Oost J, de Vos WM, Smidt H. The use of defined microbial communities to model host-microbe interactions in the human gut. Microbiol Mol Biol Rev. 2019; 83(2):e00054–18. doi: 10.1128/MMBR.00054-18 . 30867232 PMC6684003

[28] Vrancken G, Gregory AC, Huys GRB, Faust K, Raes J. Synthetic ecology of the human gut microbiota. Nat Rev Microbiol. 2019; 17(12):754–763. doi: 10.1038/s41579-019-0264-8 . 31578461

[29] Eng A, Borenstein E. Microbial community design: methods, applications, and opportunities. Curr Opin Biotechnol. 2019; 58:117–128. doi: 10.1016/j.copbio.2019.03.002 . 30952088 PMC6710113

[30] Shetty SA, Kuipers B, Atashgahi S, Aalvink S, Smidt H, de Vos WM. Inter-species metabolic interactions in an in-vitro minimal human gut microbiome of core bacteria. NPJ Biofilms Microbiomes. 2022; 8(1):21. doi: 10.1038/s41522-022-00275-2 Erratum in: NPJ Biofilms Microbiomes. 2022; 8(1):76. . 35395818 PMC8993927

[31] Medina DA, Pinto F, Ovalle A, Thomson P, Garrido D. Prebiotics mediate microbial interactions in a consortium of the infant gut microbiome. Int J Mol Sci. 2017; 18(10): 2095. doi: 10.3390/ijms18102095 . 28976925 PMC5666777

[32] van der Lelie D, Oka A, Taghavi S, Umeno J, Fan TJ, Merrell KE, et al. Rationally designed bacterial consortia to treat chronic immune-mediated colitis and restore intestinal homeostasis. Nat Commun. 2021; 12(1):3105. doi: 10.1038/s41467-021-23460-x . 34050144 PMC8163890

[33] De Giani A, Bovio F, Forcella ME, Lasagni M, Fusi P, Di Gennaro P. Prebiotic effect of Maitake extract on a probiotic consortium and its action after microbial fermentation on colorectal cell lines. Foods 2021; 10:2536. doi: 10.3390/foods10112536 . 34828817 PMC8617840

[34] De Giani A, Oldani M, Forcella M, Lasagni M, Fusi P, Di Gennaro P. Synergistic antioxidant effect of prebiotic Ginseng berries extract and probiotic strains on healthy and tumoral colorectal cell lines. Int. J. Mol. Sci. 2022b; 24(1):373. doi: 10.3390/ijms24010373 . 36613815 PMC9820163

[35] Presti I, D’ Orazio G, Labra M, La Ferla B, Mezzasalma V, Bizzaro G, et al. Evaluation of the probiotic properties of new *Lactobacillus* and *Bifidobacterium* strains and their in vitro effect. Appl Microbiol Biotechnol. 2015; 99(13):5613–26. doi: 10.1007/s00253-015-6482-8 . 25744647

[36] Watson D, O’ Connell Motherway M, Schoterman MH, van Neerven RJ, Nauta A, van Sinderen D. Selective carbohydrate utilization by lactobacilli and bifidobacteria. J Appl Microbiol. 2013; 114(4):1132–46. doi: 10.1111/jam.12105 . 23240984

[37] Mezzasalma V, Manfrini E, Ferri E, Sandionigi A, La Ferla B, Schiano I, et al. A randomized, double-blind, placebo-controlled trial: the efficacy of multispecies probiotic supplementation in alleviating symptoms of irritable bowel syndrome associated with constipation. Biomed. Res. Int. 2016; 2016:4740907. doi: 10.1155/2016/4740907 . 27595104 PMC4993960

[38] De Giani A, Sandionigi A, Zampolli J, Michelotti A, Tursi F, Labra M, et al. Effects of inulin-based prebiotics alone or in combination with probiotics on human gut microbiota and markers of immune system: a randomized, double-blind, placebo-controlled study in healthy subjects. Microorganisms 2022c; 10(6):1256. doi: 10.3390/microorganisms10061256 . 35744774 PMC9229734

[39] Déjean G, Tamura K, Cabrera A, Jain N, Pudlo NA, Pereira G, et al. Synergy between cell surface glycosidases and glycan-binding proteins dictates the utilization of specific beta(1,3)-glucans by human gut Bacteroides. mBio. 2020; 11(2):e00095–20. doi: 10.1128/mBio.00095-20 . 32265336 PMC7157763

[40] Alauzet C, Cunat L, Wack M, Lozniewski A, Busby H, Agrinier N, et al. Hypergravity disrupts murine intestinal microbiota. Sci Rep. 2019; 9(1):9410. doi: 10.1038/s41598-019-45153-8 . 31253829 PMC6599200

[41] Xie YH, Gao QY, Cai GX, Sun XM, Sun XM, Zou TH, et al. Fecal *Clostridium symbiosum* for noninvasive detection of early and advanced colorectal cancer: test and validation studies. EBioMedicine. 2017; 25:32–40. doi: 10.1016/j.ebiom.2017.10.005 . 29033369 PMC5704049

[42] Huijsdens XW, Linskens RK, Mak M, Meuwissen SG, Vandenbroucke-Grauls CM, Savelkoul PH. Quantification of bacteria adherent to gastrointestinal mucosa by real-time PCR. J Clin Microbiol. 2002; 40(12):4423–7. doi: 10.1128/JCM.40.12.4423-4427.2002 . 12454130 PMC154607

[43] Ogita T, Yamamoto Y, Mikami A, Shigemori S, Sato T, Shimosato T. Oral administration of *Flavonifractor plautii* strongly suppresses Th2 immune responses in mice. Front. Immunol. 2020; 11:379. doi: 10.3389/fimmu.2020.00379 . 32184789 PMC7058663

[44] Palframan R, Gibson G, Rastall R. Development of a quantitative tool for the comparison of the prebiotic effect of dietary oligosaccharides. Lett. Appl. Microbiol. 2003; 37:281–4. doi: 10.1046/j.1472-765x.2003.01398.x . 12969489

[45] Strati F, Pujolassos M, Burrello C, Giuffrè MR, Lattanzi G, Caprioli F, et al. Antibiotic-associated dysbiosis affects the ability of the gut microbiota to control intestinal inflammation upon fecal microbiota transplantation in experimental colitis models. Microbiome 2021; 9(1):39. doi: 10.1186/s40168-020-00991-x . 33549144 PMC7868014

[46] Forster SC, Kumar N, Anonye BO, Almeida A, Viciani E, Stares MD, et al. A human gut bacterial genome and culture collection for improved metagenomic analyses. Nat Biotechnol. 2019; 37(2):186–192. doi: 10.1038/s41587-018-0009-7 . 30718869 PMC6785715

[47] Eckburg PB, Bik EM, Bernstein CN, Purdom E, Dethlefsen L, Sargent M, et al. Diversity of the human intestinal microbial flora. Science. 2005; 308(5728): 1635–8. doi: 10.1126/science.1110591 . 15831718 PMC1395357

[48] Vaga S, Lee S, Ji B, Andreasson A, Talley NJ, Agréus L, et al. Compositional and functional differences of the mucosal microbiota along the intestine of healthy individuals. Sci Rep. 2020; 10(1): 14977. doi: 10.1038/s41598-020-71939-2 . 32917913 PMC7486370

[49] Cieplak T, Wiese M, Nielsen S, Van de Wiele T, van den Berg F, Nielsen DS. The Smallest Intestine (TSI)-a low volume *in vitro* model of the small intestine with increased throughput. FEMS Microbiol Lett. 2018; 365(21). doi: 10.1093/femsle/fny231 . 30247563

[50] Ma G, Du H, Hu Q, Yang W, Pei F, Xiao H. Health benefits of edible mushroom polysaccharides and associated gut microbiota regulation. Crit. Rev. Food Sci. Nutr. 2022; 62(24):6646–6663. doi: 10.1080/10408398.2021.1903385 . 33792430

[51] Jayachandran M, Xiao J, Xu B. A critical review on health promoting benefits of edible mushrooms through gut microbiota. Int J Mol Sci. 2017; 18(9): 1934. doi: 10.3390/ijms18091934 . 28885559 PMC5618583

[52] Sveinbjørnsson B, Rushfeldt C, Seljelid R, Smedsrød B. Inhibition of establishment and growth of mouse liver metastases after treatment with interferon gamma and beta-1,3-D-glucan. Hepatology. 1998; 27(5): 1241–8. doi: 10.1002/hep.510270509 . 9581677

[53] Adachi Y, Ohno N, Ohsawa M, Sato K, Oikawa S, Yadomae T. Physiochemical properties and antitumor activities of chemically modified derivatives of antitumor glucan "grifolan LE" from Grifola frondosa. Chem Pharm Bull (Tokyo) 1989; 37(7): 1838–43. doi: 10.1248/cpb.37.1838 . 2805163

[54] Fernandez-Julia P, Black GW, Cheung W, Van Sinderen D, Munoz-Munoz J. Fungal β-glucan-facilitated cross-feeding activities between Bacteroides and Bifidobacterium species. Commun Biol. 2023; 6(1): 576. doi: 10.1038/s42003-023-04970-4 . 37253778 PMC10229575

[55] Mitsou EK, Saxami G, Stamoulou E, Kerezoudi E, Terzi E, Koutrotsios G, et al. Effects of rich in β-glucans edible mushrooms on aging gut microbiota characteristics: an *in vitro* study. *Molecules*. (2020) 25:2806. doi: 10.3390/molecules25122806 32570735 PMC7355846

[56] Abreu NA, Taga ME. Decoding molecular interactions in microbial communities. FEMS Microbiol Rev. 2016; 40(5):648–63. doi: 10.1093/femsre/fuw019 . 27417261 PMC5007284

[57] Casal JJ, Asís SE. Natural and synthetic quinoline derivatives as anti-tuberculosis agents. Austin Tuberc Res Treat. 2017; 2(1):1007.

[58] Mithul Aravind S, Wichienchot S, Tsao R, Ramakrishnan S, Chakkaravarthi S. Role of dietary polyphenols on gut microbiota, their metabolites and health benefits. Food Res Int. 2021; 142:110189. doi: 10.1016/j.foodres.2021.110189 . 33773665

[59] Burrello C, Garavaglia F, Cribiù FM, Ercoli G, Lopez G, Troisi J, et al. Therapeutic faecal microbiota transplantation controls intestinal inflammation through IL10 secretion by immune cells. Nat Commun. 2018; 9(1):5184. doi: 10.1038/s41467-018-07359-8 . 30518790 PMC6281577

[60] Blaak EE, Canfora EE, Theis S, Frost G, Groen AK, Mithieux G. Short chain fatty acids in human gut and metabolic health. Benef Microbes. 2020; 11(5): 411–455. doi: 10.3920/BM2020.0057 . 32865024

[61] Cheung EC, Vousden KH. The role of ROS in tumour development and progression. Nat Rev Cancer. 2022; 22(5):280–97. doi: 10.1038/s41568-021-00435-0 . 35102280

[62] Busuttil RA, Garcia AM, Cabrera C, Rodriguez A, Suh Y, Kim WH, et al. Organ-specific increase in mutation accumulation and apoptosis rate in CuZn-superoxide dismutase-deficient mice. Cancer Res. 2005; 65(24):11271–5. doi: 10.1158/0008-5472.CAN-05-2980 . 16357131

[63] González-Bosch C, Boorman E, Zunszain PA, Mann GE. Short-chain fatty acids as modulators of redox signaling in health and disease. Redox Biol. 2021; 47: 102165. doi: 10.1016/j.redox.2021.102165 . 34662811 PMC8577496

[64] Tan YQ, Wang YN, Feng HY, Guo ZY, Li X, Nie XL, et al. Host/microbiota interactions-derived tryptophan metabolites modulate oxidative stress and inflammation via aryl hydrocarbon receptor signaling. Free Radic Biol Med. 2022; 184:30–41. doi: 10.1016/j.freeradbiomed.2022.03.025 . 35367341

[65] Burrello C, de Visser KE. Pulling the strings of the tumor microenvironment. Cancer Immunol Res. 2022; 10(1):4. doi: 10.1158/2326-6066.CIR-21-0977 . 34911740

[66] Campos-Perez W, Martinez-Lopez E. Effects of short chain fatty acids on metabolic and inflammatory processes in human health. Biochim Biophys Acta Mol Cell Biol Lipids. 2021; 1866(5): 158900. doi: 10.1016/j.bbalip.2021.158900 . 33571672

