# Supplementary figures and images for: Positive modulation of a new reconstructed human gut microbiota by Maitake extract helpfully boosts the intestinal environment in vitro
Source: PLoS One. 2024 Apr 11;19(4):e0301822. doi: 10.1371/journal.pone.0301822 (PMC11008829; doi:10.1371/journal.pone.0301822)

**A**

**B**

**C**


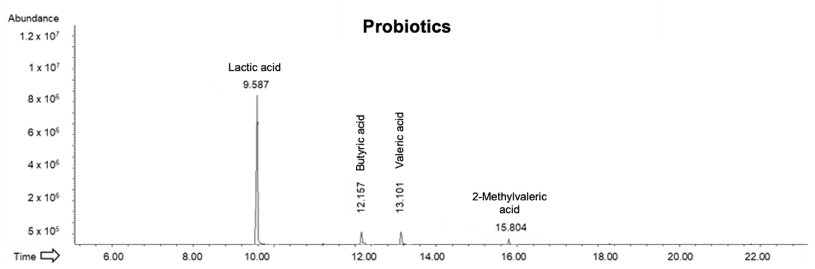

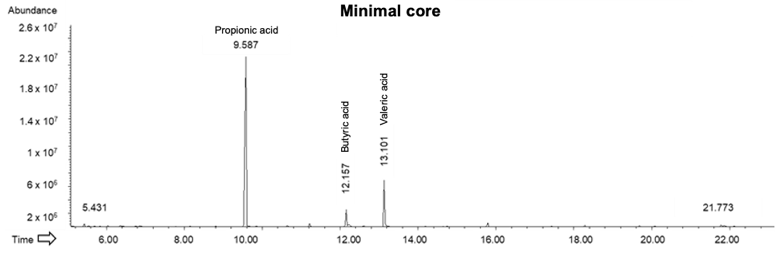

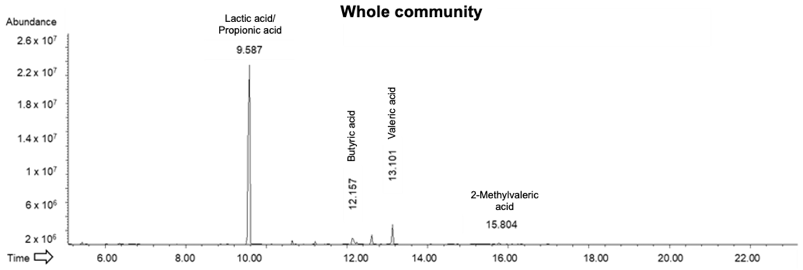

Supplement: S1 Fig — The growth is presented as OD600nm (A) and bacterial counts/mL (B) during the time. The produced metabolites are illustrated as results of GC-MSD analyses (C). (DOCX) [file pone.0301822.s001.docx]
