# Supplementary material for: Positive modulation of a new reconstructed human gut microbiota by Maitake extract helpfully boosts the intestinal environment in vitro
Source: PLoS One. 2024 Apr 11;19(4):e0301822. doi: 10.1371/journal.pone.0301822 (PMC11008829; doi:10.1371/journal.pone.0301822)
Supplement: S1 Table — (DOCX) [file pone.0301822.s003.docx]

| STRAIN | Primer code | Sequence 5’ 🡪 3’ | DNA region | Reference |
| --- | --- | --- | --- | --- |
| *L. plantarum* | Lpl2F  Lpl2R | CATTGGAACCGAACCAGTTG CGGTGTTCTCGGTTTCATTATG | 16S/23S IS | [37,38] |
| *L. acidophilus* | Lacid2F  Lacid2R | GGGCAAATCACGAACGAGTA CTTTGTTTTCGTTCGCTTCA | Pre 16S | [37,38] |
| *B. animalis* subsp. *lactis* | AnimF  AnimR | GCACGGTTTTGTGGCTGG GACCTGGGGGACACACTG | Pre 16S | [37,38] |
| *B. cellulosilyticus* | BAC_16S_F  BAC_16S_R | GGTAGTCCACACAGTAAACGATGAA  CCCGTCAATTCCTTTGAGTTTC | 16S | [39] |
| *F. plautii* | Fplautii170F  Fplautii423R | GGTCGCATGGCTCTGACT  TCATTTGTTTCGTCCCCGAC | 16S | [40] |
| *C. symbiosum* | CS_F  CS_R | GTGAGATGATGTGCCAGGC TACCGGTTGCTTCGTCGATT | 2-*hydroxyglutaryl-CoA dehydratase* | [41] |
| *E. coli* | EC_F  EC_R | CATGCCGCGTGTATGAAGAA CGGGTAACGTCAATGAGCAAA | 16S | [42] |

**Table S1**. List of the bacterial primer sets utilized in the study.
