# Supplementary material for: Positive modulation of a new reconstructed human gut microbiota by Maitake extract helpfully boosts the intestinal environment in vitro
Source: PLoS One. 2024 Apr 11;19(4):e0301822. doi: 10.1371/journal.pone.0301822 (PMC11008829; doi:10.1371/journal.pone.0301822)
Supplement: S2 Table — (DOCX) [file pone.0301822.s004.docx]

| Antibodies | source | identifier |
| --- | --- | --- |
| CD3 – APCCy7 | Tonbo | 25-0038-T100 |
| CD4 – PeCy7 | Invitrogen | 25-0047-42 |
| cd1d:PBS57 Tetramer-PE | NIH Tetramer core facility | - |
| TIM-3 – BV786 | Biolegend | 345032 |
| PD-1 – BV510 | BD | 563076 |
| CD25 – FITC | Biolegend | 302604 |
| CD127 – SB600 | eBioscience | 63-1278-42 |
| IL-10 – PerCP | eBioscience | 46-7108-42 |
| E4BP4 – APC | Miltenyi Biotec | 130-11-249 |
| FOXP3 – eF450 | eBioscience | 48-5773-82 |

**Table S2**. Directly conjugated antibodies used for flow cytometry experiments.
