# Supplementary material for: Positive modulation of a new reconstructed human gut microbiota by Maitake extract helpfully boosts the intestinal environment in vitro
Source: PLoS One. 2024 Apr 11;19(4):e0301822. doi: 10.1371/journal.pone.0301822 (PMC11008829; doi:10.1371/journal.pone.0301822)
Supplement: S3 Table — (DOCX) [file pone.0301822.s005.docx]

| Target | Purified antibody | biotin-conjugated antibody | standard | streptavidin-hrp |
| --- | --- | --- | --- | --- |
| IL-6 | Biolegend | Biolegend | Biolegend | mabtech |
| IL-10 | Biolegend | Biolegend | Biolegend | mabtech |
| IL-8 | BD | BD | Biolegend | mabtech |

**Table S3**. List of ELISA reagents.
