# Supplementary material for: Positive modulation of a new reconstructed human gut microbiota by Maitake extract helpfully boosts the intestinal environment in vitro
Source: PLoS One. 2024 Apr 11;19(4):e0301822. doi: 10.1371/journal.pone.0301822 (PMC11008829; doi:10.1371/journal.pone.0301822)
Supplement: S4 Table — (DOCX) [file pone.0301822.s006.docx]

| gene | Primer code | Sequence 5’ 🡪 3’ | source | cat. number |
| --- | --- | --- | --- | --- |
| RPL32 | F  R | GCCCAAGATCGTCAAAAAGAGA  TCCGCCAGTTACGCTTAATTT | Sigma | - |
| SOD1 | F  R | GGTGGGCCAAAGGATGAAGAG  CCACAAGCCAAACGACTTCC | Sigma | - |
| AHR | F  R | ACATCACCTACGCCAGTCG  CGCTTGGAAGGATTTGACTTGA | Sigma | - |
| NQO1 | F  R | GAAGAGCACTGATCGTACTGGC  GGATACTGAAAGTTCGCAGGG | Sigma | - |
| CYP1A1 | F  R | TCGGCCACGGAGTTTCTTC  GGTCAGCATGTGCCCAATCA | Sigma | - |
| OCLN_1 | - | - | Qiagen | QT00081844 |
| REG3G_1 | - | - | Qiagen | QT01030596 |
| TJP1_1 (ZO-1) | - | - | Qiagen | QT00077308 |

**Table S4**. Primer list for human cell lines.
